# G-quadruplex binding properties of a potent PARP-1 inhibitor derived from 7-azaindole-1-carboxamide

**DOI:** 10.1038/s41598-021-83474-9

**Published:** 2021-02-16

**Authors:** Sabrina Dallavalle, Loana Musso, Roberto Artali, Anna Aviñó, Leonardo Scaglioni, Ramon Eritja, Raimundo Gargallo, Stefania Mazzini

**Affiliations:** 1 Department of Food, Environmental and Nutritional Sciences (DEFENS), University of Milan (Università Degli Studi Di Milano), Milan, Italy; 2 Scientia Advice di Roberto Artali, 20832 Desio, MB Italy; 3 Institute for Advanced Chemistry of Catalonia (IQAC), CSIC, Networking Center on Bioengineering, Biomaterials and Nanomedicine (CIBER-BBN), Barcelona, Spain; 4 Department of Chemical Engineering and Analytical Chemistry, University of Barcelona, Barcelona, Spain

**Keywords:** Drug discovery, Chemistry

## Abstract

Poly ADP-ribose polymerases (PARP) are key proteins involved in DNA repair, maintenance as well as regulation of programmed cell death. For this reason they are important therapeutic targets for cancer treatment. Recent studies have revealed a close interplay between PARP1 recruitment and G-quadruplex stabilization, showing that PARP enzymes are activated upon treatment with a G4 ligand. In this work the DNA binding properties of a PARP-1 inhibitor derived from 7-azaindole-1-carboxamide, (2-[6-(4-pyrrolidin-1-ylmethyl-phenyl)-pyrrolo[2,3-b]pyridin-1-yl]-acetamide, compound **1**) with model duplex and quadruplex DNA oligomers were studied by NMR, CD, fluorescence and molecular modelling. We provide evidence that compound **1** is a strong G-quadruplex binder. In addition we provide molecular details of the interaction of compound **1** with two model G-quadruplex structures: the single repeat of human telomeres, d(TTAGGGT)_4_, and the c-MYC promoter Pu22 sequence. The formation of defined and strong complexes with G-quadruplex models suggests a dual G4 stabilization/PARP inhibition mechanism of action for compound **1** and provides the molecular bases of its therapeutic potential.

## Introduction

Cancer remains a leading cause of death worldwide. A majority of anticancer drugs induce cell death through DNA binding and damage. Advances in the molecular biology of cancer have identified key mechanisms involved in DNA repair induced by chemotherapeutic drugs indicating that cancer cells overexpress DNA repair pathways as mechanisms of drug resistance^1^. Thus, blocking these pathways has emerged as an attractive approach for cancer therapy^2^.

Polyadenylation diphosphate ribose transferases (poly ADP-ribose polymerases, PARPs) are DNA repair enzymes. The superfamily of PARPs consists of six different enzymes: PARP-1, PARP-2, PARP-3, PARP-4/VPARP, tankyrase-1 and tankyrase-2. These enzymes are involved in various cellular functions, including cell cycle regulation, transcription, and DNA damage repair. PARP-1 is currently the best characterized enzyme in this group. Its intervention takes place early in the steps of the DNA repair process, as this enzyme binds to and is activated by DNA nicks^3,4^. Upon poly-ADP ribosylation, the enzyme repairs breaks in single-strand DNA through a base excision repair pathway.

Inhibition of PARP activity prevents repair activity, hampering DNA strand rejoining, consequently generating permanent single/double-strand breaks, which in turn trigger cell death.

**Figure 1:**
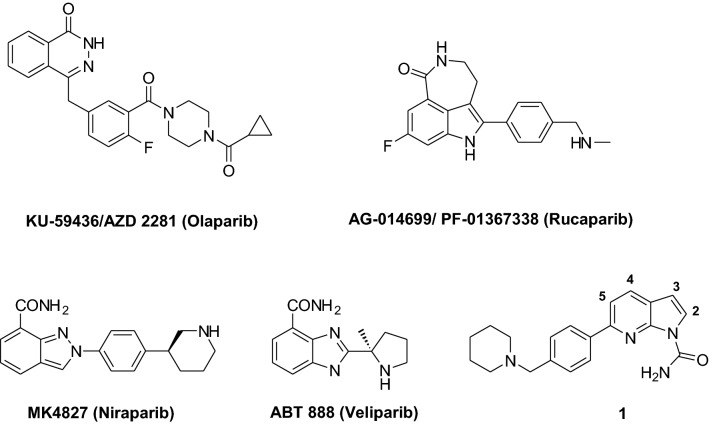
Structures of PARP inhibitors.

Currently, numerous PARP inhibitors are under investigation as chemo- and radio-sensitizers for cancer treatment^5–7^. In the last three decades, a large number of PARP-1 inhibitors have been reported, including Veliparib, Olaparib, Rucaparib and Niraparib (Fig. 1). The last three and talazoparib have been approved and are in clinical use for treatment of ovarian cancer^7^. Some others are in advanced clinical trials as single agents or in combination with DNA-damaging drugs. In fact, it has been demonstrated that the inhibition of PARPs potentiates the activity of DNA-targeting agents^8–10^.

G-quadruplexes (G4s) are non-canonical nucleic acids secondary structures that may form in G-rich sequences under physiological conditions. Their structural building block is the G-quartet, a planar array of 4 guanines paired through Hoogsteen bonds. G4s play an important role in several key cellular processes, including gene transcription, chromatin epigenetics and DNA recombination. For this reason, G-quadruplex DNAs are considered novel therapeutic targets for anticancer drug development. G4 DNA is found in key regulatory regions of the cell such as promoters of proto-oncogenes (c-MYC, BCL-2, c-Kit) and telomeres. Human telomeres were among the first discovered and characterized sequences adopting G quadruplex structures^11^. Telomere maintenance and safeguard have a critical role in carcinogenesis. The telomerase access to the telomeres can be blocked by forming and stabilizing the structures of the G-quadruplex of telomeric ends, eventually leading to the inhibition of the enzyme catalytic activity^12^. In promoters, G-quadruplexes are considered molecular switches that are able to turn transcription processes on and off in association with transcriptional proteins that may facilitate their folding. The possibility of modulate this on/off mechanisms as well as the telomerase action has triggered the development of large number of G-quadruplex small molecule ligands to stabilize these G4 DNA structures as promising anticancer drugs^13^.

Recent studies have revealed a close interplay between PARP1 recruitment and G-quadruplex stabilization, showing that PARP enzymes are activated upon treatment with a G4 ligand^14^. It has been demonstrated that the concomitant exposure to PARP inhibitors prevented repairing of DNA breaks, which were induced by G4 stabilization. This eventually led to increased chromosome abnormalities and inhibition of tumour cell growth both in vitro and in tumour xenografts.

The impact of these findings is broad, as they reveal the key role of PARP-1 in G-quadruplex related DNA damages. Another complex and perhaps indirect correlation between G4 stabilization and PARP inhibition is shown by CX-5461, an inhibitor of RNA polymerase I transcription of ribosomal RNA genes, which stabilizes G4^15–17^. Sanij et al. have recently found that the combination of CX-5461 and a PARPi exacerbates replication stress and DNA damage of HR-proficient high grade serous ovarian cancer (HGSOC) cells and significantly improves the regression of HR-deficient HGSOC-PDX tumours in vivo^18^.

The inhibition of RNA polymerase I by CX-5461 is shared by other compounds, such as BMH-21 and analogues, which have been found to interact with G4 quadruplexes^17^.

To exploit the synergistic effect of G4 stabilization and PARPs inhibition, multiple-target compounds with a dual G4/PARP mechanism of action have been designed^19^.

An intriguing approach would be finding among well known PARP inhibitors an efficient G4 stabilizer.

To the best of our knowledge, only a few groups explored the binding mode of PARP inhibitors to DNA. Yang and coworkers investigated the binding interaction of two PARP inhibitors in clinical trials, ABT88813^20^ and Niraparib (MK-4827)^21^ with calf thymus deoxyribonucleic acid (ctDNA). The exploration by theoretical and experimental techniques showed that ABT-888 bound to DNA by a partial intercalation mode. On the other hand, theoretical calculations suggested that ABT-888 preferably bound to the DNA groove. Conversely, intercalation was reported to be the main DNA binding mode for Niraparib. This compound was said to preferentially intercalate into the A–T-rich rather than into the C–G-rich regions of DNA.

These studies stimulated our interest towards a deeper investigation of the specific DNA binding mode of PARP inhibitors, which currently remains quite elusive.

We have recently designed and synthesized 7-azaindole-1-carboxamides as a new class of PARP-1 inhibitors^22^. A selected compound **1** (2-[6-(4-pyrrolidin-1-ylmethyl-phenyl)-pyrrolo[2,3-b]pyridin-1-yl]-acetamide, Fig. 1) showed a significant inhibition of the enzyme and ability to bypass the multidrug resistance mediated by Pgp. In antitumor activity studies, Compound **1** exhibited in vivo higher activity against the MX1 human breast carcinoma growth in nude mice than the reference compound Olaparib in terms of tumor volume inhibition. Treatment was well tolerated, as all the treated animals survived without significant weight losses^22^.

In this paper we report a fluorescence, CD, NMR and molecular modelling study focused on the interaction of **1** with the single repeat sequence of human telomeres, d(TTAGGGT)_4_^23^, and with the G-quadruplex found in the c-MYC promoter Pu22 sequence, whose overexpression is one of the most common aberration in a wide range of human tumors. In addition, the interaction with duplex oligonucleotides, d(CGTACG)_2_ and with d(AAGAATTCTT)_2_ was also investigated by ^1^H and ^31^P NMR spectroscopy.

## Results and discussion

### ^1^H and ^31^P NMR of 1 with double helix B-DNA d(CGTACG)_2_ and d(AAGAATTCTT)_2_

The NMR spectra of both the self-complementary oligonucleotides d(CGTACG)_2_ (“CG”) and d(AAGAATTCTT)_2_ (“AATT”) display signals in a region ranging from 12 to-13.5 ppm, which are characteristic of the NH imino protons of CG and AT base pairs. The presence of these signals confirms that both oligomers adopt, in Na^+^ solution, a double helix conformation. For this reason, they were used as models for CG- and AT-rich sequences, respectively.

**Figure 2:**
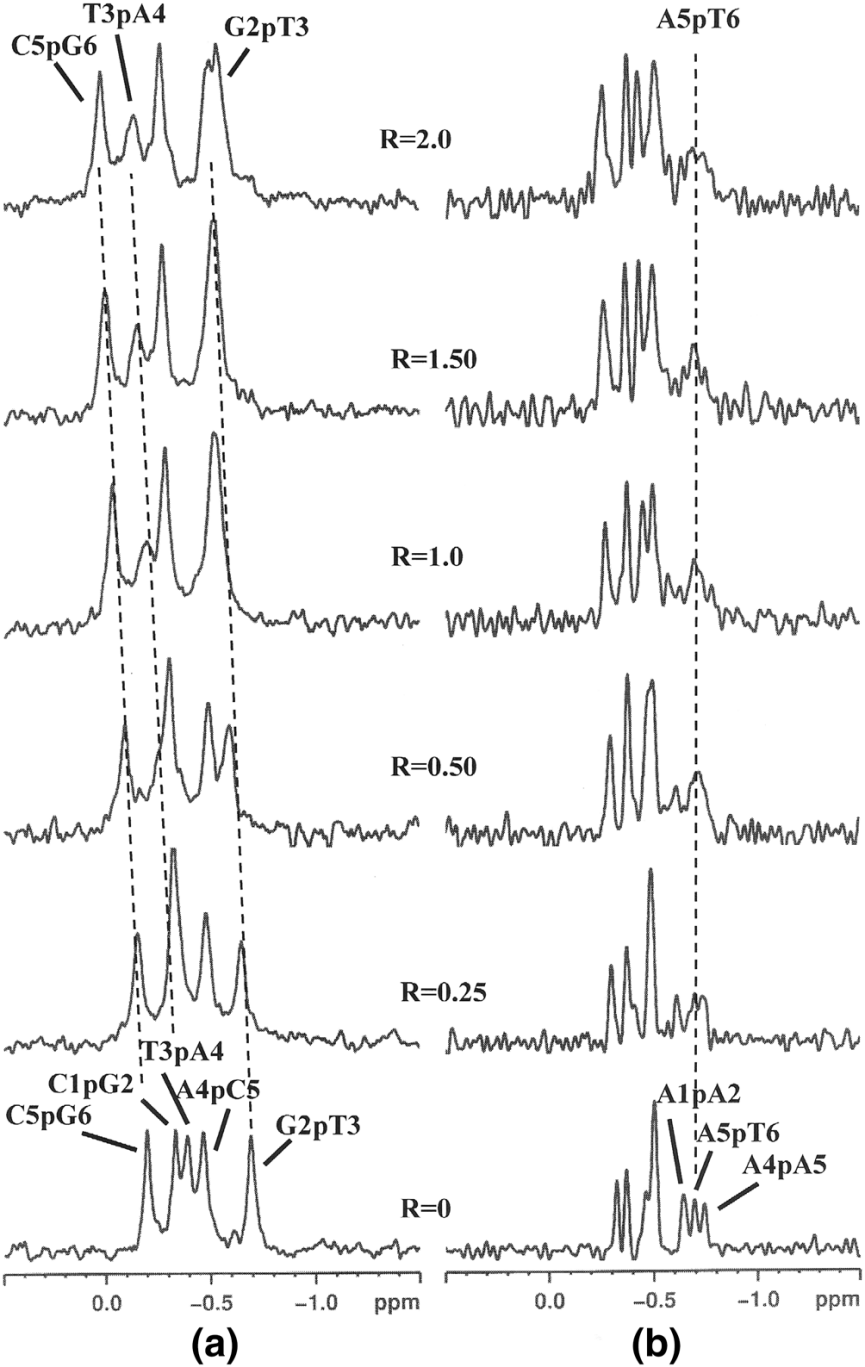
^1^H-decoupled ^31^P NMR spectra of (**a**) d(CGTACG)_2_ and (**b**) d(AAGAATTCTT)_2_ with **1** at 15 °C in H_2_O/D_2_0 (9:1), 0.1 M NaCl and 10 mM sodium phosphate buffer, pH = 7.0, at different R = [drug]/[DNA] ratios.

The phosphorus spectra of “CG” with **1** showed a low-field shift variation of G2pT3, T3pA4 and C5pG6 signals (Fig. 2a; Table 1). It is known that 31P resonance is a sensitive probe to detect changes in the phosphoribose chain of the oligonucleotides due to the intercalation process; in fact, the chemical shift variation of the ^31^P resonances reflects a deformation at the level of P–O(5′) and P–O(3′) bonds. As a consequence of the intercalation of a ligand into the oligonucleotide double helix, the *alfa* = O(3′)–P–O(5′)–C(5′) and *zeta* = C(3′)–O(3′)–P–O(5′) angles change from a *gauche*, *gauche* conformation (− 60° and − 90°) to a *gauche*, *trans* conformation (− 60°,  + 180°). This conformational change is usually associated with a low-field shift up to 1.0–2.5 ppm for ^31^P resonances^24,25^. In our case the lower Δδ values (− 0.2 ppm) found for G2pT3, T3pA4 and C5pG6 suggest that **1** is bound to the oligonucleotide, however through a partial intercalation binding mode, at these three sites, with a possible exchange among them. The addition of **1** to “AATT” induced insignificant chemical shift variation of the phosphate signals in the ^31^P NMR spectra (Fig. 2b; Table 1). This is the proof that an intercalation process did not occur. Nevertheless, the A1pA2, A5pT6 and A4pA5 signals became very broad even at R = 0.25. The ^1^H NMR titration with **1** gave different results for the two oligonucleotides. Broadening of the imino NH signals with no relevant chemical shift variation was observed for “AATT” resonances, whereas only the H1′ anomeric protons belonging to “AATT” tract were slightly perturbed (Δδ =  −028/ −0.11 ppm) (Table S1). A line broadening was observed for the aromatic protons of A5 and T6, while the H2 proton of A4, which lies in the minor groove, splitted into two signals just at R = 0.25. This suggests the formation of both a free and a bound species; for R ≥ 1.0 the free species disappears (Figure S1).

**Table 1:** ^31^P chemical shift assignments of phosphate in the free oligonucleotides and in the complex with **1**^a,b^. Oligonucleotide “CG” corresponds to duplex d(CGTACG)_2_ and “AATT” is duplex d(AAGAATTCTT)_2_.

“CG”	δ (ppm)	Δδ^c^	“AATT”	δ (ppm)	Δδ^c^
C1pG2	− 0.27	+ 0.07	A1pA2	broad	n.d
G2pT3	− 0.49	+ 0.21	A2pG3	− 0.25	+ 0.07
T3pA4	− 0.13	+ 0.26	G3pA4	− 0.5^d^	0.00
A4pC5	− 0.54	− 0.07	A4pA5	broad	n.d
C5pG6	+ 0.04	+ 0.24	A5pT6	broad	n.d
−			T6pT7	− 0.45^d^	+ 0.05
−			T7pC8	− 0.38	+ 0.07
−			C8pT9	− 0.50^d^	0.00
−			T9pT10	− 0.38	0.00

^a^Measured at 15 °C in ppm (δ) from external DSS. Solvent H_2_O–D_2_O (90:10 v/v), of 0.1 M NaCl and 10 mM sodium phosphate buffer solution, pH = 7.0.

^b^R = 2.0.

^c^Δδ = δ_bound_ − δ_free_.

^d^The assignment might be interchanged.

The addition of **1** to a solution of “CG” presented a significant up-field chemical shift variation either for imino, aromatic or anomeric protons (Figure S2). The most affected were the protons of the G2 and C5 units, in line with the interaction at these sites. The unique exception was represented by the T3 anomeric proton (Δδ + 0.19 ppm) (Table S1). A possible explanation of this deshielding effect is that the intercalation of the ligand at G2pT3 places the piperidine moiety in the minor groove at the level of T3.

2D-NOESY experiments did not show intermolecular Nuclear Overhauser Effect (NOE) contacts between the ligand and both oligonucleotides, probably due to a weak interaction or to a rapid exchange between different binding sites. Therefore, it was not possible to build a model for the complexes with **1**.

Overall, these results show that **1** partially intercalates with a CG-rich sequence, whereas it gives a slight external interaction with an AT-rich sequence. In conclusion, the interaction of **1** with double helix oligonucleotides can be considered not relevant.

However, these findings cannot rule out an interaction with G-quadruplex DNA structures, whose stabilization has been found to activate PARP-1 enzyme^14^. Thus, we focused our study on the interaction of compound **1** with G-quadruplex structures of telomeres and proto-oncogenes.

### Interaction of 1 with telomere d(TTAGGGT)_4_ quadruplex

**Figure 3:**
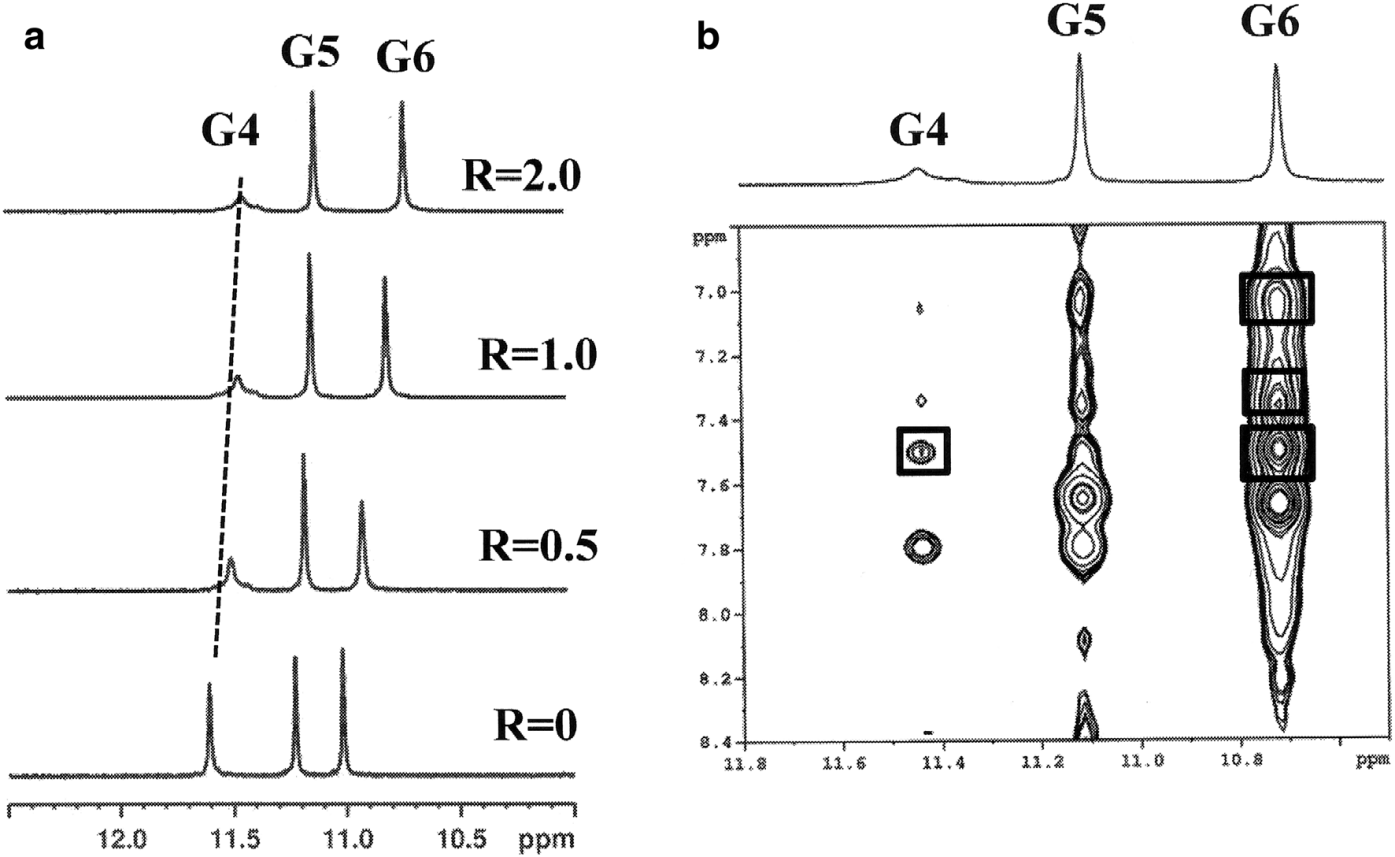
Imino proton region of (**a**) 1D NMR titration spectra of d(TTAGGGT)_4_ with **1** at 25 °C at different R = [drug]/[DNA] ratios; (**b**) expanded region of 2D NOESY spectrum of **1**/ d(TTAGGGT)_4_ complex at R = 2.0. The boxes display the intermolecular NOE interaction of NH imino protons with pyrrole moiety and aromatic protons of **1**.

The titration of the oligonucleotide solution with **1** induced significant line broadening of G4 NH signal and less pronounced broadening of the aromatic resonances of the three guanines (Fig. 3a). An upfield shift variation was observed for the imino, aromatic and anomeric proton signals of all units, except for T7 (Table S2). Also the resonances of the ligand gave an upfield shift. They were partially overlapped to the signals of the oligonucleotide, however they were identified by a TOCSY experiment at 7.02 and 7.49 ppm (pyrrole moiety), at 7.09 and 7.38 ppm (phenyl and pyridine moieties). NOE contacts were found for A3 H8 and G4 NH with the pyrrole protons. Other NOE interactions involved NH and H8 of G6 with both pyrrole and aromatic protons of the ligand (Table 2) (Fig. 3b). These results indicate that **1** binds to the G-quadruplex at two sites: between A3 and G4 and over G6.

**Table 2:** Intermolecular NOE in the complex of **1** with d(TTAGGGGT)_4_.

	NOE	d (Å)^a^
**A3G4 binding site**		
**1**	(TTAGGGT)_4_	
H2	A3H8	5.01
H3	A3H8	3.61
H aromatic/Phe	A3H8	4.37, 4.60
H2	G4H1	4.26
**G6T7 binding site**		
H2	G6H1	4.97
H3	G6H1	3.93
H aromatic/Phe	G6H1	3.69
H aromatic/H3	G6H8	4.57, 4.79

Acquired at 25 °C in H_2_O–D_2_O (90:10 v/v), 25 mM K-phosphate buffer, 150 mM KCl, 1 mM EDTA, pH 6.7

^a^Distances obtained by molecular modelling of the complex.

**Figure 4:**
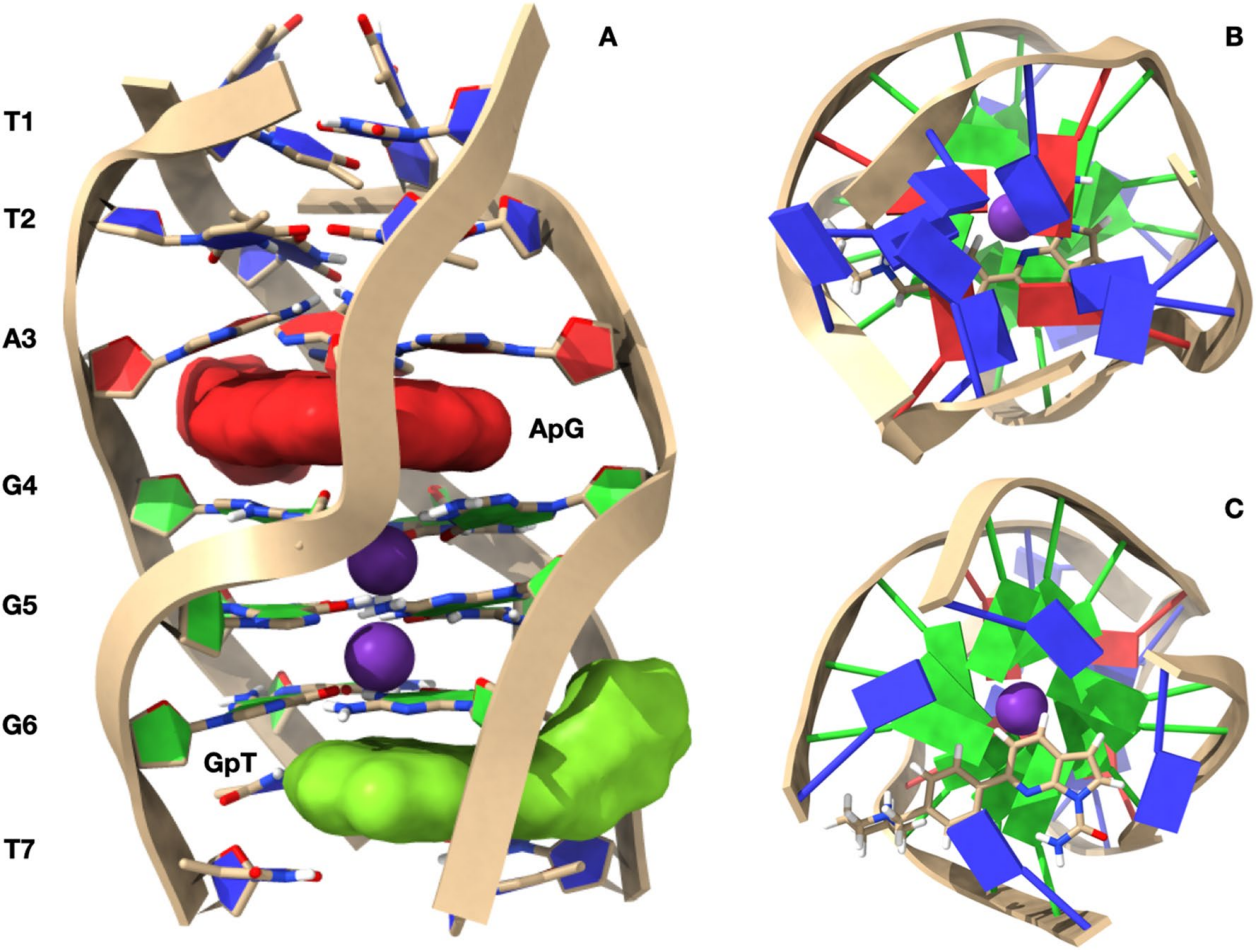
Representation of the complexes at the A–G and G–T intercalation sites, obtained by Molecular Docking and optimized by Molecular Dynamics. (**A**) Side view of d(TTAGGGT)_4_ with the nucleotides represented in stick and filled rings: Adenine in red, Guanine in green and Thymine in blue. The ligand is represented by its solvent accessible surface (SAS), red for the ligand at A–G and green for the ligand at the G–T intercalation site. The optimized conformations of the ligand are represented in **B** for the complex at A–G and in **C** for the complex at G–T. Potassium ions are represented by their Van der Waals spheres (K^+^ in purple), while the ligand is depicted in stick and coloured following the CPK code. The nucleotides are represented as filled plates: Adenine in red, Guanine in green and Thymine in blue. Molecular graphic was obtained with UCSF ChimeraX, https://www.cgl.ucsf.edu/chimerax/.

The interaction of **1** with the quadruplex d(TTAGGGT)_4_ was also investigated using the molecular docking technique, followed by optimization via molecular dynamics (MD). The molecule was docked at sites A3–G4 and G6–T7. In both cases the ligand does not adopt a center-symmetrical stacking interaction with the upper and lower tetrads, but it is rather displaced towards two of the four residues (Fig. 4).

At the AG intercalation site the 7-azaindole portion of the ligand is inserted between A3 and G4. In this intercalation site the complex is also stabilized by a hydrogen bond between the NH_2_ group of A3 and the aromatic nitrogen of the pyrrole[2,3-b]pyridine moiety, at a distance of 2.98 Å. The piperidine ring is oriented outward from the quadruplex, with the quaternary nitrogen forming a hydrogen bond with N_7_ of A3 (2.63 Å), a cation–π interaction with G4, and a ionic interaction with OP_2_A3 (Fig. 4B).

At the GT intercalation site, the 7-azaindole moiety of the ligand gives rise to π–π stacking interactions with G6 and T7. In this case, the complex is stabilized by a hydrogen bond between O_4_ T7 and the CONH_2_ group of the ligand, at a distance of 2.36 Å. The -CONH2 group itself is locked in position by an intra-molecular hydrogen bond with the pyridine nitrogen (2.24 Å). Again, the piperidine ring points outward from the quadruplex, with the quaternary nitrogen forming a hydrogen bond with N_3_ of G6 (2.58A) and an ionic bond with OP_1_T7 (Fig. 4C).

The best docked conformations of the complexes at the A3G4 and G6T7 intercalation sites are in good agreement with the reported NOE contacts (Table 2).

### Interaction of 1 with G-quadruplex Pu22T14T23 sequence

**Figure 5:**
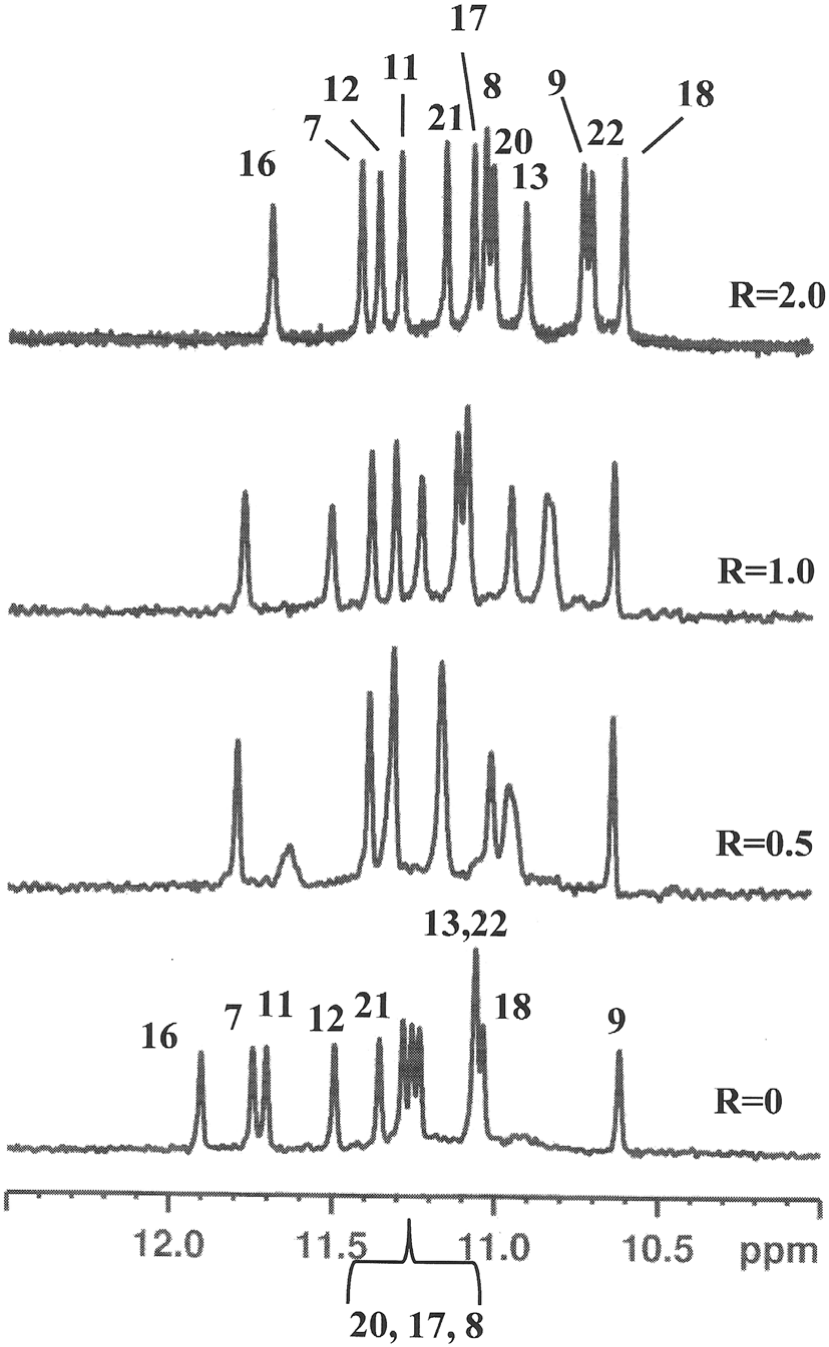
Imino proton region of 1D NMR titration spectra of Pu22T14T23 with **1** at 25 °C at different R = [drug]/[DNA] ratios.

Pu22T14T23 gave high quality spectra in K^+^ solution in comparison with wild-type sequence^17,26^. For this reason, it has been chosen as a good model to study ligand-quadruplex interaction. ^1^H NMR titration experiments were performed by adding increasing amounts of ligand to Pu22T14T23 solution, with ratios R = [ligand]/[DNA] ranging from 0 to 2.5. The NMR data indicated a single G-quadruplex conformation for the complex, with each proton showing a single chemical shift value and NOEs characteristic of the three G-tetrad stacked structure (Table S3 and Table S4). The titration experiment proved a chemical shift perturbation of the imino protons (Fig. 5). The largest perturbations were observed for G18 and G22 (3′-end) and for G7, G11, G16 and G20 (5′-end). Less relevant perturbations were observed for the imino protons of G8, G12, G17 and G21, in the central guanine tetrad. No relevant chemical shift variation was observed for the residues located in the loops of the G-quadruplex, such as A15, T14 and T19 (Table S3). This excludes the interaction of **1** with the groove. These findings suggest that **1** stacks on the 3′ and 5′ sites of Pu22T14T23.

**Figure 6:**
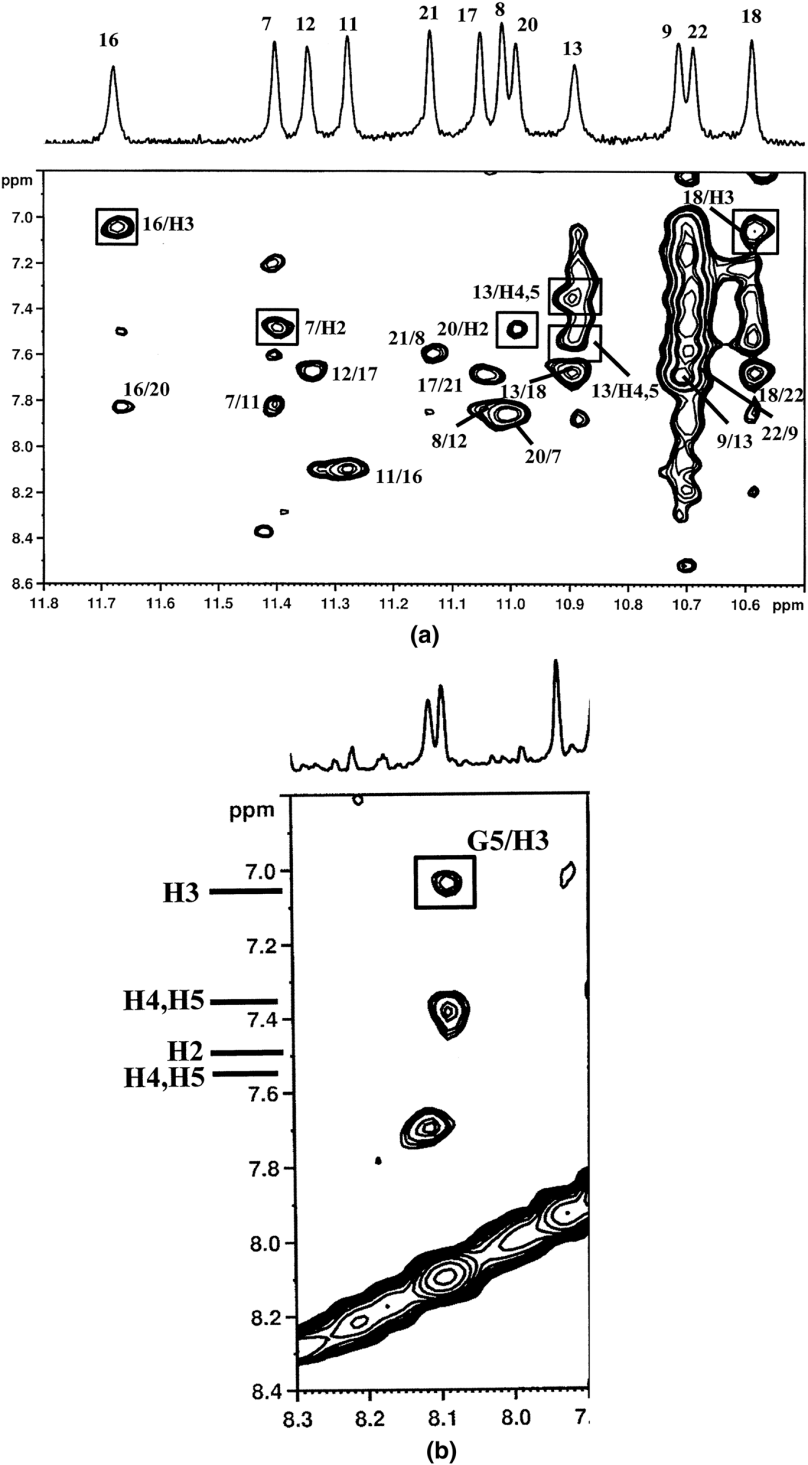
Selected region of 2D NOESY spectrum of **1**/ Pu22T14T23 complex at R = 2.0. (**a**) The boxes display the intermolecular NOE interaction between NH imino protons with pyrrole moiety H2 and H3 and aromatic protons (H4 and H5) of **1**; the inter-residue NOE interactions of NH/aromatic protons of the guanines in the tetrads are also indicated. (**b**) The box displays the intermolecular NOE interaction between H8 of G5 residue with pyrrole H3 of **1**.

NOE contacts were found between the ligand and the imino NH protons of G13, G18 (3′-end), G7, G16 and G20 (5′-end) of the quadruplex (Fig. 6). The aromatic protons of the ligand could not be unambiguously assigned because of their overlapping. However, the resonances of H2 and H3 of the pyrrole moiety were identified by TOCSY experiments at 7.49 and 7.04 ppm, respectively. These protons show NOE contacts with the imino NH of G16 and G18 (H3) and with G7 and G20 (H2) (Table 3). In addition an NOE interaction was detected between H8G5 and H3 of pyrrole moiety. Other resonances of the ligand were identified at 7.38 and 7.55 ppm, showing a NOE interaction with NH G13 of the quadruplex. No NOEs between the ligand and the flanking chains were detected.

**Table 3:** Intermolecular NOEs in the complexes of **1** with Pu22T14T23^a^ and distances from modelling.

		d (Å)^b^
**3**′**-binding site NOE**		
**1**	Pu22T14T23	
H4, H5	G13H1	5.09, 5.59
H3	G18H1	5.50
**5**′**-binding site NOE**		
H2	G7H1	5.55
H3	G16H1	3.74
H3	G5H8	4.52
H2	G20H1	4.89

^a^Acquired at 25 °C in H_2_O–D_2_O (90:10 v/v), 25 mM K-phosphate buffer, 70 mM KCl, 1 mM EDTA, pH 6.9

^b^Distances obtained by molecular modelling of the complex.

**Figure 7:**
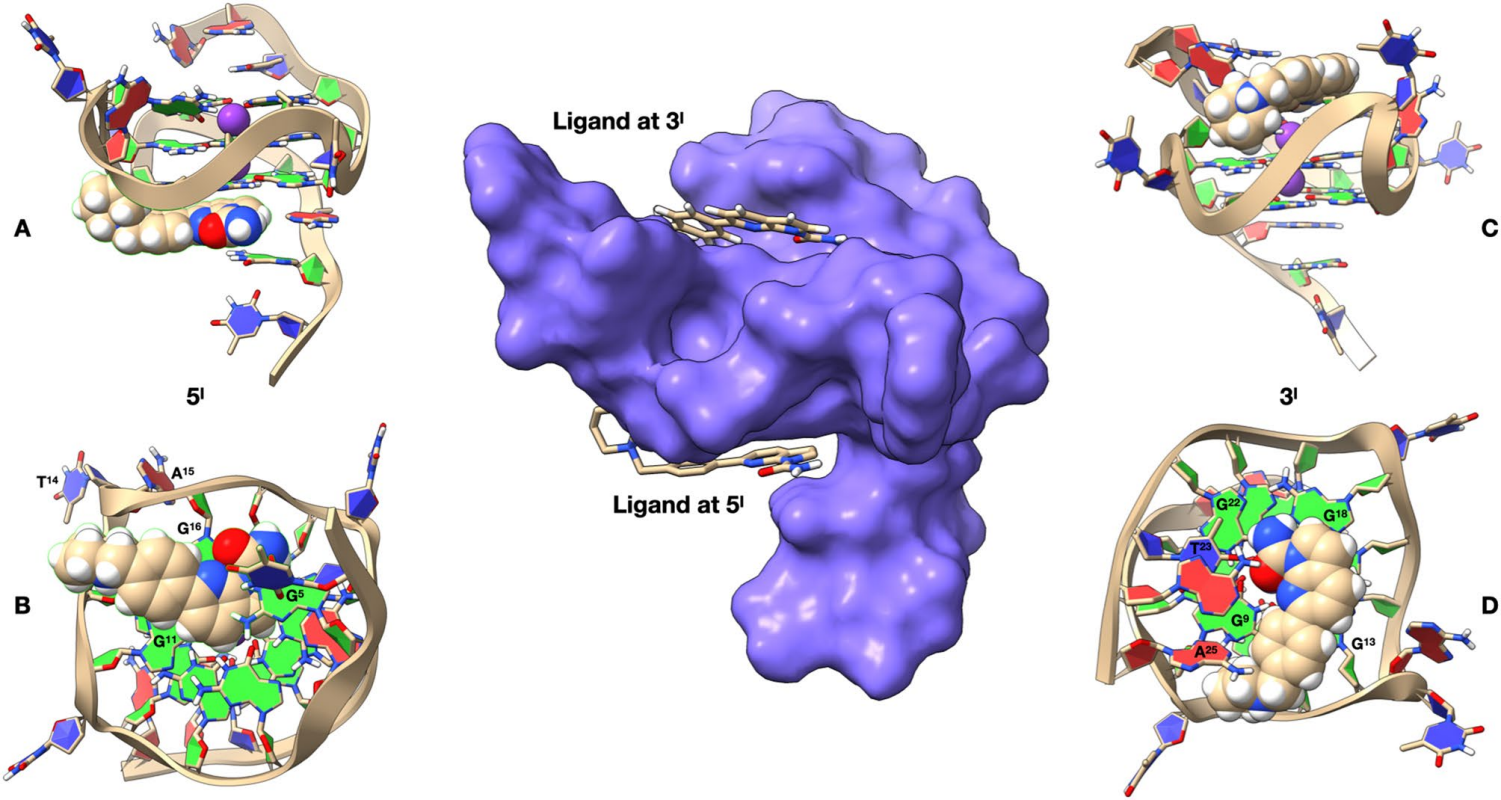
Representation of the complexes with **1** at 5′-end and 3′-end, obtained by Molecular Docking and optimized by Molecular Dynamics. At the center of the figure Pu22T14T23, represented by its solvent accessible surface (SAS, in blue) and complexed with **1** (represented in stick) at both 5′-end and 3′-end. On the left, lateral (**A**) and bottom (**B**) representation of **1** conformation at the 5′-end complex, while on the right we can see the lateral (**C**) and top (**D**) representation of **1** conformation at the 3′-end complex. Ligand and potassium ions are represented by their Van der Waals spheres (ligand colored in CPK, K^+^ in purple), while the nucleotide units of Pu22T14T23 are represented as filled rings: Adenine in red, Guanine in green and Thymine in blue. Nucleotides capable of interacting with the ligand are labelled in (**B**) and (**D**). Molecular graphic was obtained with UCSF ChimeraX, https://www.cgl.ucsf.edu/chimerax/.

In order to obtain a three-dimensional model congruent with the NOE contacts we performed a molecular docking experiment, followed by MD calculations (Fig. 7). At 5′-end, **1** is stabilized by an extensive network of π–π interactions involving the underlying 5′-end G-tetrad, with the pyrrole[2,3-b]pyridine moiety located near the center of the tetrad (Fig. 7a, b). The above cited aromatic rings interact with the π systems of G5, G11 and G16. They are held in place by the cation-π interaction between the potassium ion and the pyrrole moiety (4.96 Å). Also the central phenyl ring creates π–π interactions with G11 and G16, while the piperidine ring is oriented outside the system, towards G11, T14 and A15, without giving rise to observable interactions. The -CONH2 group is coplanar with the pyridine aromatic system and positioned above G16, without giving noteworthy interactions with Pu22.

At 3′-end, the complex is also stabilized by a dense network of π–π interactions involving all the guanines of the tetrad: specifically, the pyrrole[2,3-b]pyridine moiety interacts with the π systems of G18 and G13 (Fig. 7c, d). The phenyl group of 1 forms π–π interactions with the G13 unit as well. This is complemented by a further π–π interaction with the G9 aromatic system. The CONH_2_ group is oriented towards G22 and T23, forming two hydrogen bonds: one with O6G22 (2.84 Å) and the other with O4T23 (3.02 Å). The piperidine ring is oriented outside the system, in the area between G9, G13 and A25, and does not present particular interactions with Pu22. In both 3′-end and 5′-end positions, the piperidine ring is arranged along the main groove of Pu22, with a docking score difference in favour of the complex in 3′ (ΔE = 5.77 kcal/mol). The best docked conformations of the complexes at 5′ and 3′ are in good agreement with the reported NOE contacts (Table 3).

The flanking chains at the two terminals in the complex, even though no NOE interactions with the ligand were detected, showed significant chemical shift variations, especially in the segment T23-A24-A25 at the 3′-end. Many resonances move upfield: T23 Me (Δδ =  −0.28), T23 H6 and H1′ (Δδ =  −0.11 and −0.30); H8 of A24 and A25 (Δδ =  −0.38 and − 0.32). Other resonances move low-field, the most significant being the anomeric protons H1′′of A25 and A24 (Δδ + 0.57 and + 0.25, respectively), and a slight deshielding of G9 NH (Δδ + 0.10). This finding suggests that the three units T23, A24 and A25 do not prevent the binding. The ligand, positioned on the 3′-end tetrad, changes the architecture of the tail of this terminal. Actually, the structure of the free nucleotide Pu22T14T23 shows that A25 folds back to form a base pair with T23, thus protecting the external 3′-end G-tetrad. The entry of the ligand breaks the Hoogsteen-type H-bond between T23 and A25^27^, pushing away T23 toward the top of G22, thus experiencing the stacking effect of this guanine. This is highlighted by the up-field shift of T23 protons, being also in line with the model. The A25 unit is no more folded over the G9 aromatic moiety, as in the free nucleotide^27^. This justifies the deshielding of G9 NH and of the anomeric H1′ protons of both A25 and A24. The aromatic portion of these units is slightly shielded. This may be explained with the increased flexibility of the tail. At 5′-end the ligand induces small conformational changes as shown by the slightly low-field shift of the T4, G5 and A6 aromatic protons (Δδ =  +0.09, − 0.15 ppm), which indicates that also this tail is pushed away from the tetrad.

Similar experiments were also performed using ABT-888 as a ligand, in order to find possible interactions with Pu22T14T23 and to compare the results with those above described for **1**. The titration of Pu22T14T23 with ABT888 did not show any chemical shift variation (Figure S3) and no NOE contact was found in the NOESY spectra. Consequently, we must conclude that the Pu22T14T23 quadruplex of the c-MYC promoter is not a target for ABT-888.

### CD and fluorescence studies

**Figure 8:**
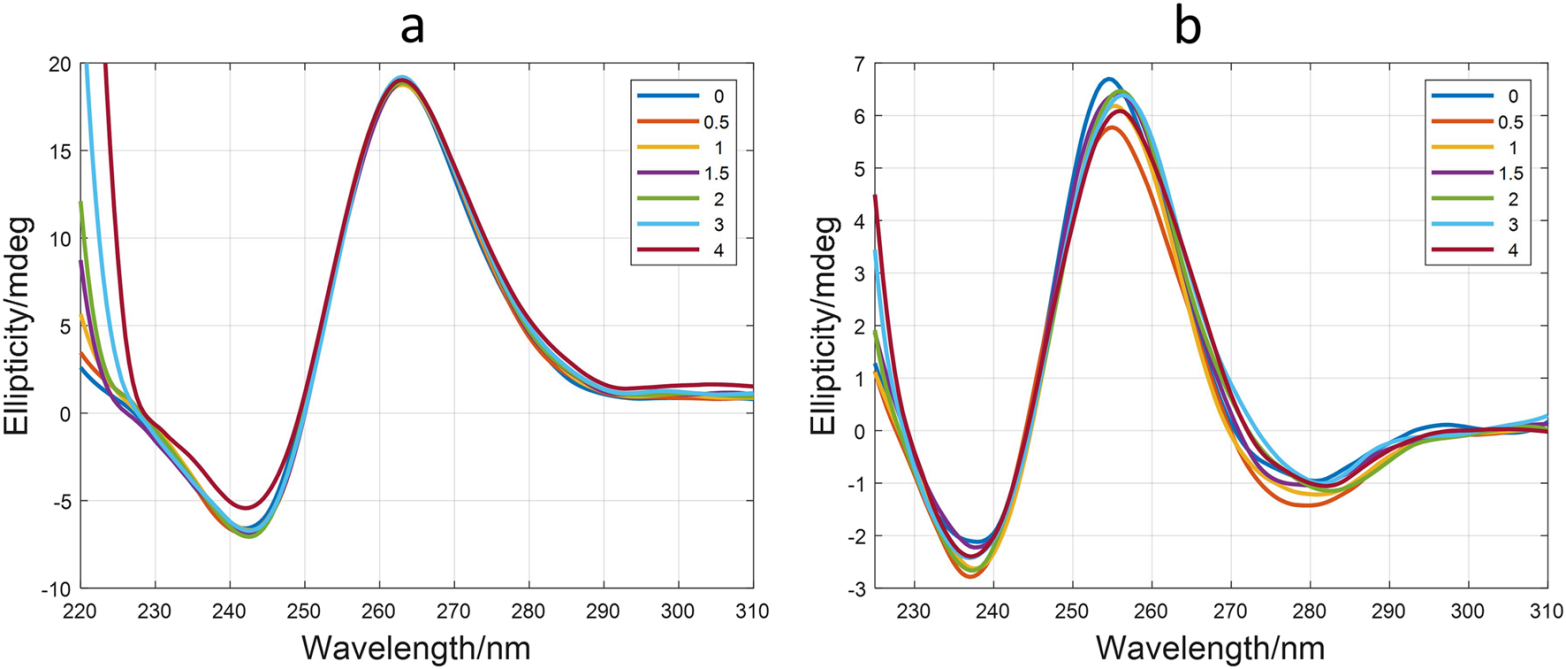
CD-monitored titrations of Pu22T14T23 (**a**) and d(TTAGGGT)4 (**b**) with ligand **1**. In both cases, the numbers in inset indicate the ratio ligand:DNA. Experimental conditions were 25 mM phosphate buffer and 70 mM KCl (Pu22T14T23) or 150 mM KCl (HT), 15 °C.

Both DNA G-quadruplex sequences, d(TTAGGGT)_4_ and Pu22T14T23, used for NMR investigation, form stable parallel G-quadruplex structures at the experimental conditions used for CD and fluorescence studies (Fig. 8). The intramolecular G-quadruplex formed by Pu22T14T23 showed a Tm value around 85 °C (Figure S4), which indicated a high thermal stability of this structure. On the other hand, the human telomeric sequence formed an intermolecular G-quadruplex structure. Therefore, the kinetics of the folding/unfolding process depends so strongly on DNA concentration that determination of the right Tm value would need an extremely small heating rate, due to the presence of hysteresis. Consequently, the midpoint of the transition determined in the conditions used in this work should be named as T_1/2_^28^, being its value 50 °C for d(TTAGGGT)_4_.

CD-monitored titrations of both G-quadruplexes with the ligand **1** were carried out to determine potential structural and, global changes due to the interaction. Titrations were carried out at 15 °C, where both G-quadruplex structures are the major species. No clear changes were observed that could be related to dramatic structural modifications of the G-quadruplex (Fig. 8). Upon addition of the ligand, no variations could be observed in Tm value of the G-quadruplex formed by Pu22T14T23, in agreement with the high stability of this structure.

The fluorescence spectra of ligand **1** in presence of both G-quadruplex sequences are shown in Figure S5. The ligand showed strong fluorescence in potassium phosphate buffer solution without oligonucleotides. The addition of the G-quadruplex structures induced a decrease of the fluorescence signal intensity. From the titration curves, an estimation of the stoichiometry and the binding constants (Kb) relative to the interaction were done with the EQUISPEC program, which is based on the multivariate analysis of the whole spectra measured along the titration.

In both cases, the Kb values obtained for the interaction of ligand **1** with Pu22T14T23 and d(TTAGGGT)_4_ considering a 1:1 stoichiometry are in the order of 10^6.1^ M^−1^, which suggests a relatively strong interaction between this ligand and both structures. It is known that forward titrations of a ligand with increasing concentrations of DNA favours the formation and detection of the 1:1 complex, whereas reverse titrations favour the formation of complexes with higher stoichiometries^29^. In a similar way to the NMR studies, an additional reverse titration was carried out, where the Pu22T14T23 sequence was titrated with ligand **1** (Figure S6). In this case, a 1:2 (DNA:ligand) stoichiometry (overall Kb value equal to 10^12.8^ M^−2^) fitted better the experimental fluorescence data than the 1:1 stoichiometry.

Alternatively, titration curves were studied in PBS buffer obtaining a Kb value for Pu22T14T23 similar to that obtained in K buffer. However, the binding constant for d(TTAGGG)_4_ is in the order of 10^5.1^ M^−1^ (Figure S7).

## Conclusions

In this study we have investigated the specific DNA binding mode of a PARP-1 inhibitor (**1**) derived from 7-azaindole-1-carboxamide. The results provide evidence that **1** lacks the typical features of DNA intercalators. In fact, the interaction with a model of duplex DNA, as d(CGTACG)_2_, showed a partial intercalation mode at three different sites, whereas there is an external slight interaction with d( AAGAATTCTT)_2,_ a model of AT-rich sequence.

On the contrary, **1** binds both the G-quadruplexes, d(TTAGGGT)_4,_ a model of human telomere sequence, and Pu22T14T23, a model of the c-MYC promoter Pu22 sequence. In the first case, **1** is located between A3 and G4 units and over the G6 residue, while it forms a 2:1 complex with Pu22 quadruplex, with the two molecules located over the external tetrads at 5′ and 3′-end. In both cases, the Kb values obtained for the interaction of ligand **1** with Pu22T14T23 and d(TTAGGGT)_4_ are in the order of 10^6.1^ M^−1^, which indicates a significant interaction between this ligand and both structures. The strong interaction between G-quadruplex model sequences and compound **1** validates its potential therapeutic action, based on a synergistic action of PARP-1 inhibition with G-quadruplex affinity.

## Methods

### Reagents

The NMR samples of self-complementary oligonucleotides d(CGTACG)_2_ and d(AAGAATTCTT)_2_ were prepared at concentration 0.30 mM. Both sequences adopt, in presence of 0.1 M NaCl and 10 mM sodium phosphate buffer solution, pH = 7.0, a B-DNA double helix conformation. The NMR sample of Pu22T14T23 d(T G A G G G T G G G T A G G G T G G G T A A) was prepared at concentration 0.34 mM in H_2_O/D_2_O (9:1) in the presence of 25 mM KH_2_PO_4_, 70 mM KCl, pH 6.9. d(TTAGGGT)_4_ was prepared at a 0.25–0.40 mM in G-quadruplex concentration range, in H_*2*_O/D_2_O (9:1) containing 25 mM KH_2_PO_4_, 150 mM KCl and 1 mM EDTA (pH 6.7).

The oligonucleotide samples were heated to 85 °C for 1 min and then cooled at room temperature overnight. **1** was dissolved in DMSO-*d*_*6*_ at concentration of 27 mM.

### Instruments and procedures

The NMR spectra were recorded on a Bruker AV600 spectrometer operating at a frequency of 600.10 MHz and 242.94 MHz, for 1H, and 31P nuclei respectively. NMR titrations were performed at by adding increasing amounts of ligand to the DNA at different ratio R = [drug]/[DNA] from R = 0 to R = 3.0. The ^1^H and ^31^P assignments for the free oligonucleotides d(CGTACG)_2_ and d(AAGAATTCTT)_2_ have previously been reported^30,31^. Proton resonance assignments of the free d(TTAGGGT)_4_ and Pu22T14T23 sequences were performed on the basis of previous assignments^17^. The protons of the double helix and G-quadruplex oligonucleotides in the complexes were assigned using standard procedures by NOESY and TOCSY experiments (Table S1 and Table S2). Ligand protons were assigned by an integrated series of 2D experiments such as ROESY, TOCSY and COSY (Table S3). Phase sensitive NOESY spectra of the complexes were acquired at 25 °C and 15 °C in TPPI mode, with 2048 × 1024 complex FIDs. Mixing times ranged from 50 to 400 ms. TOCSY spectra were acquired with the use of a MLEV-17 spin-lock pulse (60 ms total duration). All spectra were transformed and weighted with a 90° shifted sine-bell squared function to 4 K x 4 K real data points.

### CD and fluorescence experiments

CD spectra were recorded on a Jasco J-810 spectropolarimeter equipped with a Peltier temperature control unit (Seelbach, Germany). The DNA solution (Pu22T14T23 or d(TTAGGGT)_4_ was transferred to a covered cell and ellipticity was recorded with a heating rate of approximately 0.4 °C·min^−1^. Simultaneously, CD spectra were recorded every 5 °C from 210 to 320 nm. The spectrum of the buffer was subtracted. Each sample was allowed to equilibrate at the initial temperature for 30 min before the melting experiment began. In all experiments, the concentration of DNA was kept constant (2 µM) whereas the concentration of the considered ligands was increased. The medium consisted of 25 mM KH_2_PO_4_ and 70 mM (Pu22T14T23) or 150 mM (d(TTAGGGT)_4_) KCl^32^.

Molecular fluorescence spectra were measured with an JASCO FP-6200 spectrofluorimeter. The temperature was controlled by means of a water bath. The fluorescence spectra were acquired using a quartz cuvette with a 10-mm path length. In the fluorescence measurements, both the excitation and emission slits were 10 nm, and the scan speed was 250 nm/min. Measurements were taken at 308 nm excitation wavelength. The medium consisted of 25 mM phosphate buffer (pH 6.9) and 70 mM KCl or PBS. In all experiments, the concentration of ligand was kept constant (3 µM), whereas the concentration of the considered DNA sequence was increased. The determination of the stoichiometries and the calculation of the binding constants was done from the fluorescence data recorded along titrations of ligands with DNAs by using the EQUISPEC program^33^. This program is based on the multivariate analysis of the whole spectra measured along the titration.

### Molecular modelling studies

The ligand **1** that was the object of this study was optimized as previously described^17^ while the coordinates for the Pu22T14T23 and d(TTAGGGT)_4_ starting models were obtained from the NMR structure deposited in the Protein Data Bank (accession code: 2L7V for Pu22T14T23 and 1NZM for d(TTAGGGT)_4_)^26,34^. The GROMACS package^35^ with a modified version of the 53A6 GROMOS force field^36^ was used to perform energy minimizations and molecular modeling calculations, while molecular docking experiments were conducted using the AutoDock 4.2 software^37^. The molecular docking calculations were performed using the Lamarckian Genetic Algorithm^38^, and the AutoDock Toolkit (ADT)^39^ was used to further process the ligand and the Pu22T14T23 and d(TTAGGGT)_4_ models. In ADT, the Gasteiger–Marsili charges^40^ were added to the ligand, while the phosphorus atoms in the DNA were parameterized using the Cornell parameters. The solvation parameters were added to the system by means of the Addsol utility of AutoDock. For each docking run, the initial population consisted of 100 randomly placed individuals, with a maximum number of 250 energy evaluations and a mutation rate of 0.02, a crossover rate of 0.80, and an elitism value of 1. For the local search, 250 independent docking runs were carried out for the ligand by applying the so-called pseudo-Solis and Wets algorithm with a maximum of 250 iterations per local search. The system in the actual docking process was represented by grid maps calculated with Autogrid, centered between the two K+ ions and with a grid dimensions of 80 × 80 × 80 Å (spacing of 0.01 Å). The docking results were scored by using an in-house version of the simpler intermolecular energy function based on the Weiner force field, and the results differing by less than 1.0 Å in positional root-mean-square deviation (rmsd) were clustered together and represented by the most favorable free energy of binding. The best poses obtained in the docking phase were equilibrated through 5.0 ns of molecular dynamics using the OpenCL version of the GROMACS package running on a dual-Xeon workstation (8 core) equipped with an NVIDIA GPU containing about 5000 CUDA cores. The boxes were generated by placing the systems in the centre of a box with boundaries at 2.0 nm apart from all atoms. 3′ and 5′ terminal nucleotide topologies were modified according to Ricci et al.^41^ counterions (K+ ions) were random placed and SPC water molecules were added to the systems. The full solvated systems were simulated through 100 ps of position restrained molecular dynamics, followed by a heating ramp of short (100 ps) consecutive simulations at 50, 100, 150, 200, 250, and 300 K. The production simulations consisted of 5 ns of partially restrained MD at 310 K (time step of 0.002 ps). Constraints were calculated using the Lincs^42^ and SETTLE^43^ algorithms, while Lennard–Jones interactions were calculated using a two-range switch interaction (cut-off radius of 0.9 and 1.1 nm). A Berendsen thermostat was applied^44^, (coupling time of 0.1 ps) and the electrostatic interactions were calculated using PME^45,46^, (Coulomb cut-off radius of 1.2 nm).

Molecular graphics in Figs. 4 and 7 obtained with UCSF ChimeraX, developed by the Resource for Biocomputing, Visualization, and Informatics at the University of California, San Francisco, with support from National Institutes of Health R01-GM129325 and the Office of Cyber Infrastructure and Computational Biology, National Institute of Allergy and Infectious Diseases^47,48^.

## Supplementary information

Supplementary information.
