# Supplementary material for: G-quadruplex binding properties of a potent PARP-1 inhibitor derived from 7-azaindole-1-carboxamide
Source: Sci Rep. 2021 Feb 16;11:3869. doi: 10.1038/s41598-021-83474-9 (PMC7887208; doi:10.1038/s41598-021-83474-9)
Supplement: Supplementary file 1 — Supplementary information. [file 41598_2021_83474_MOESM1_ESM.docx]

**G-quadruplex binding properties of a potent PARP-1 inhibitor derived from 7-azaindole-1-carboxamide.**

**Sabrina Dallavalle^1^, Loana Musso^1^, Roberto Artali^2^, Anna Aviñó^3^, Leonardo Scaglioni^1^, Ramon Eritja^3^, Raimundo Gargallo^4^, Stefania Mazzini^1*^**

^1^Department of Food, Environmental and Nutritional Sciences (DEFENS), University of Milan (Università degli Studi di Milano), Milan, Italy.

^2^Scientia Advice di Roberto Artali, 20832 Desio, MB, Italy

^3^Institute for Advanced Chemistry of Catalonia (IQAC), CSIC, Networking Center on Bioengineering, Biomaterials and Nanomedicine (CIBER-BBN), Barcelona, Spain.

^4^Department of Chemical Engineering and Analytical Chemistry, University of Barcelona, Barcelona, Spain.

**Contents:**

| Figure S1 | Aromatic and anomeric protons region of the 1D NMR titration spectra of d(AAGAATTCTT)_2_ with **1** at 15 °C in H_2_O/D_2_0 (9:1), 0.1 M NaCl and 10 mM sodium phosphate buffer, pH = 7.0, at different R = [drug]/[DNA] ratios. |
| --- | --- |
| Figure S2 | Aromatic and anomeric protons region of the 1D NMR titration spectra of d(CGTACG)_2_ with **1** at 15 °C in H_2_O/D_2_0 (9:1), 0.1 M NaCl and 10 mM sodium phosphate buffer, pH = 7.0, at different R = [drug]/[DNA] ratios. |
| Figure S3 | Imino proton region of 1D NMR titration spectra of Pu22T14T23 with ABT888 at 25 °C at different R = [drug]/[DNA] ratios. |
| Figure S4 | Determination of melting temperature of Pu2T14T23 in 25 mM phosphate buffer and 70 mM KCl. |
| Figure S5 | Spectra recorded along the titrations of ligand **1** with Pu22T14T23 (a) and d(TTAGGGT)_4_ (b) monitored with molecular fluorescence spectroscopy. Numbers in inset indicate the DNA:ligand ratio. Measured (blue symbols) and calculated (red lines) fluorescence signal at 384 nm considering a 1:1 model for Pu22T14T23 (c) and d(TTAGGG)_4_ (d). In both cases, the initial concentration of ligand was 3 micromolar, 25 mM phosphate buffer and 70 mM KCl, 20^o^C. |
| Figure S6 | Spectra recorded along the titration of Pu22T14T23 with ligand **1** monitored with molecular fluorescence spectroscopy (a). Numbers in inset indicate the ligand:DNA ratio. Measured (blue symbols) and calculated (red lines) fluorescence signal at 384 nm considering a 1:2 (DNA:ligand) model (b). The initial concentration of DNA was 2.4 micromolar, 25 mM phosphate buffer and 70 mM KCl, 20^o^C. |
| Figure S7 | Spectra recorded along the titrations of ligand **1** with Pu22T14T23 (a) and d(TTAGGG)_4_ (b) in PBS monitored with molecular fluorescence spectroscopy. Numbers in inset indicate the ligand:DNA ratio. Measured (blue symbols) and calculated (red lines) fluorescence signal at 384 nm considering a 1:1 model for Pu22T14T23 (c) and d(TTAGGG)_4_ (d). In both cases, the initial concentration of ligand was 3 micromolar, PBS buffer 20^o^C. |
| Table S1 | Selected ^1^ H chemical shift values for the complex of **1** with d(AAGAATTCTT)_2_ and d(CGTACG)_2_ |
| Table S2 | Selected ^1^ H chemical shift values for the complex of **1** with d(TTAGGGT)_4_ |
| Table S3 | Selected ^1^ H chemical shift values for the complex of **1** with Pu22T14T23. |
| Table S4 | Inter-residue NOE interactions of Pu22T14T23 in the complex with **1** |

**
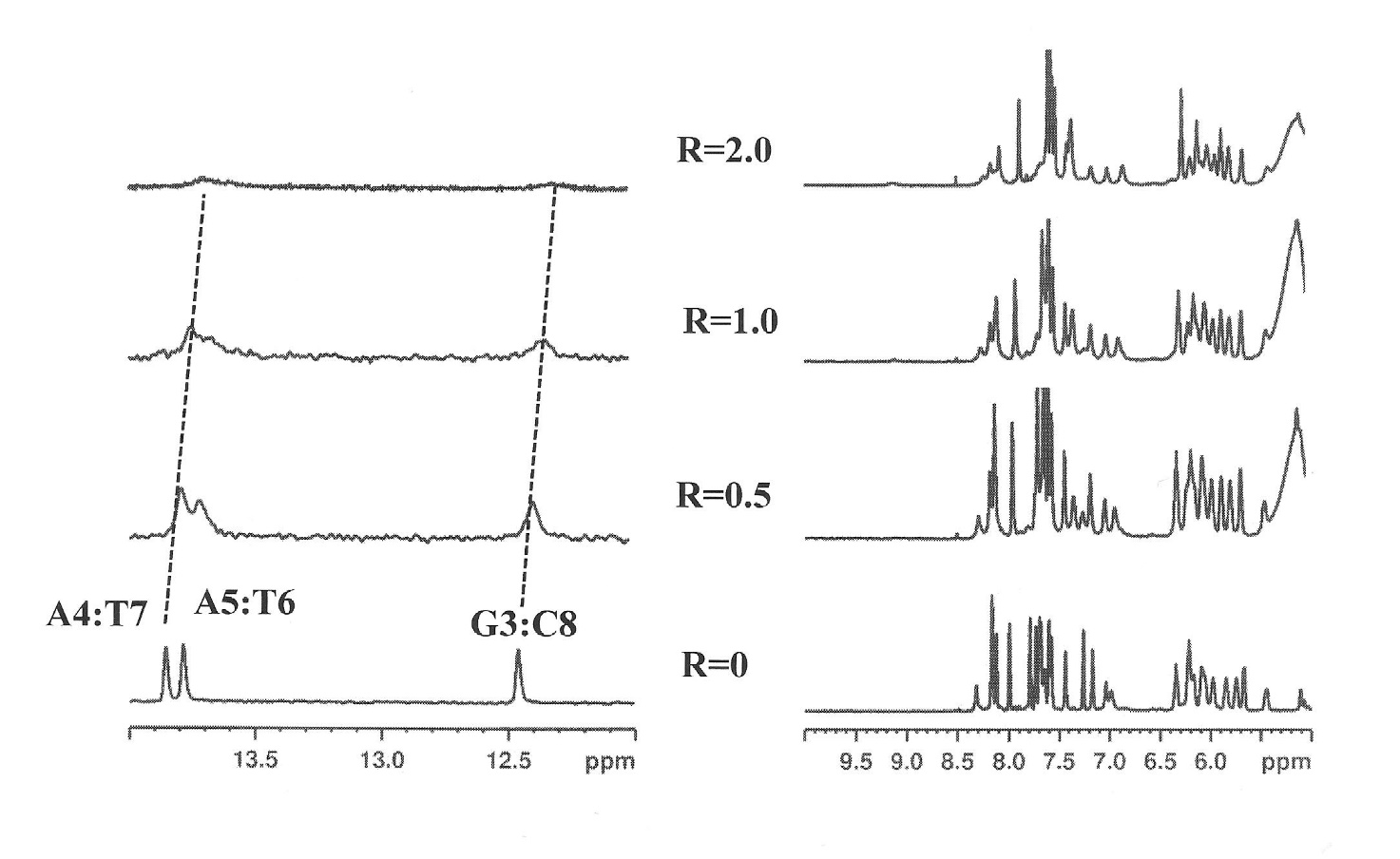
**

**Figure S1.** Aromatic and anomeric protons region of the 1D NMR titration spectra of d(AAGAATTCTT)_2_ with **1** at 15 °C in H_2_O/D_2_0 (9:1), 0.1 M NaCl and 10 mM sodium phosphate buffer, pH = 7.0, at different R = [drug]/[DNA] ratios.


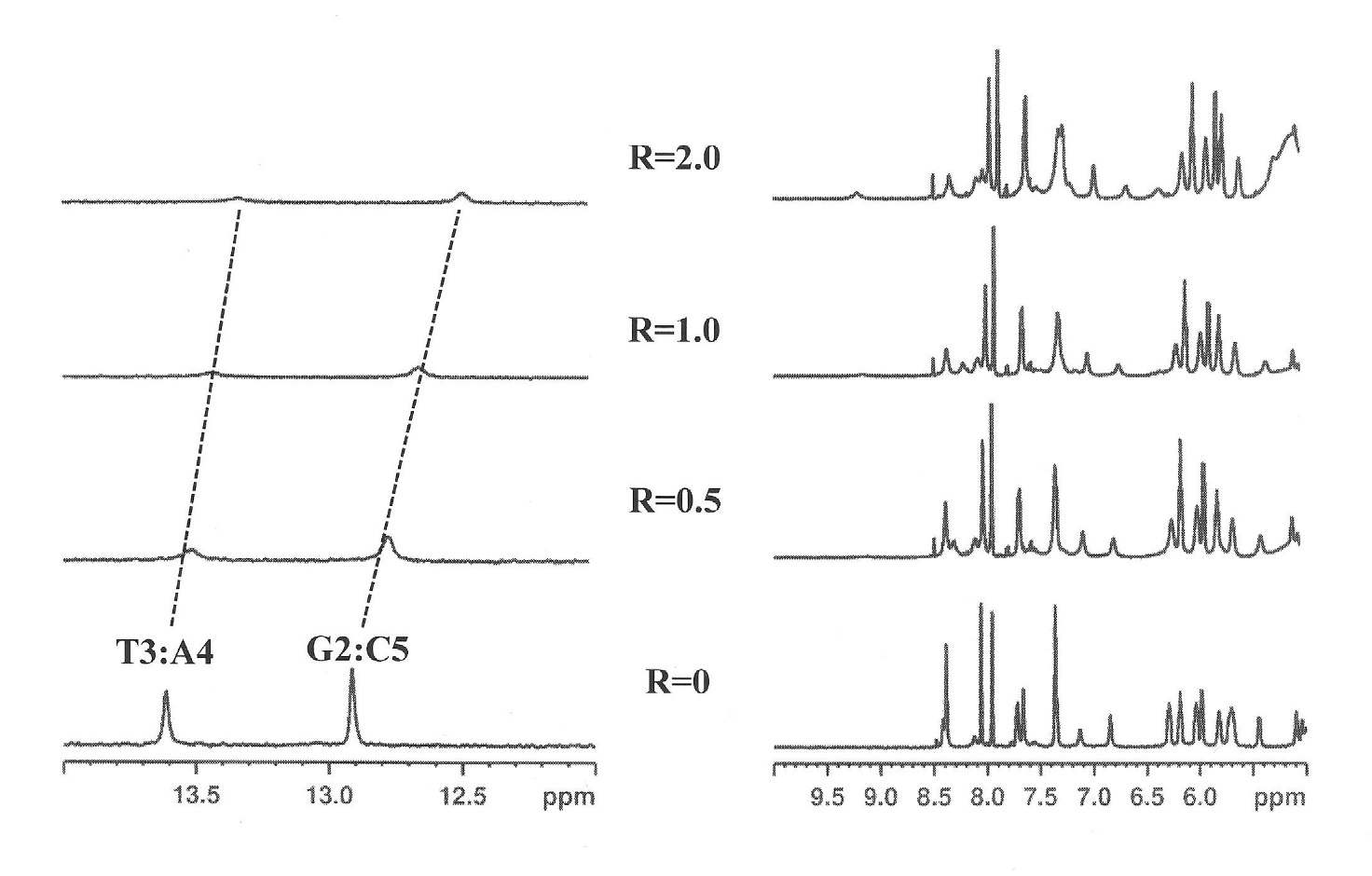


**FigureS2.** Aromatic and anomeric protons region of the 1D NMR titration spectra of d(CGTACG)_2_ with **1** at 15 °C in H_2_O/D_2_0 (9:1), 0.1 M NaCl and 10 mM sodium phosphate buffer, pH = 7.0, at different R = [drug]/[DNA] ratios.


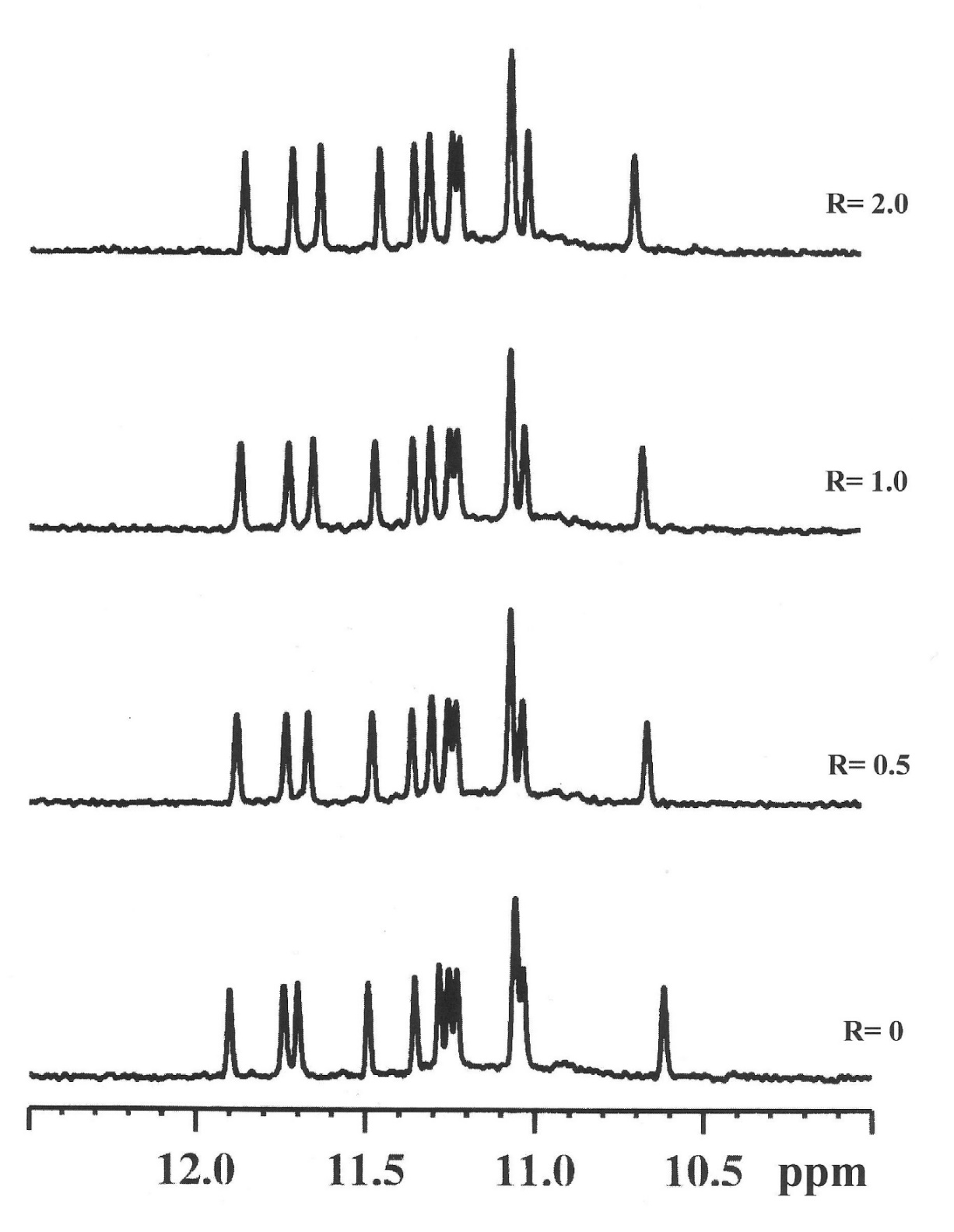


**Figure S3.** Imino proton region of 1D NMR titration spectra of Pu22T14T23 with ABT888 at 25 °C at different R = [drug]/[DNA] ratios.

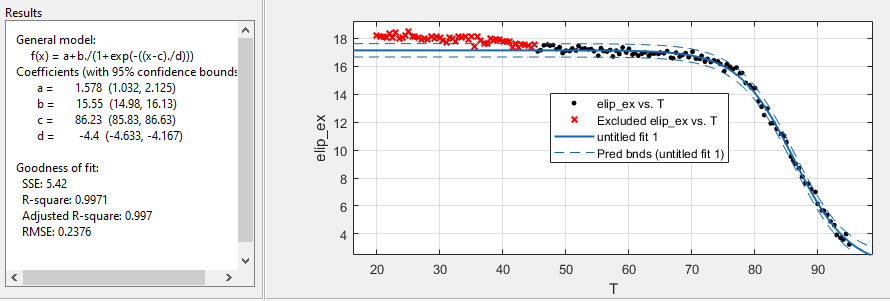


**Figure S4.** Determination of melting temperature of Pu2T14T23 in 25 mM phosphate buffer and 70 mM KCl.


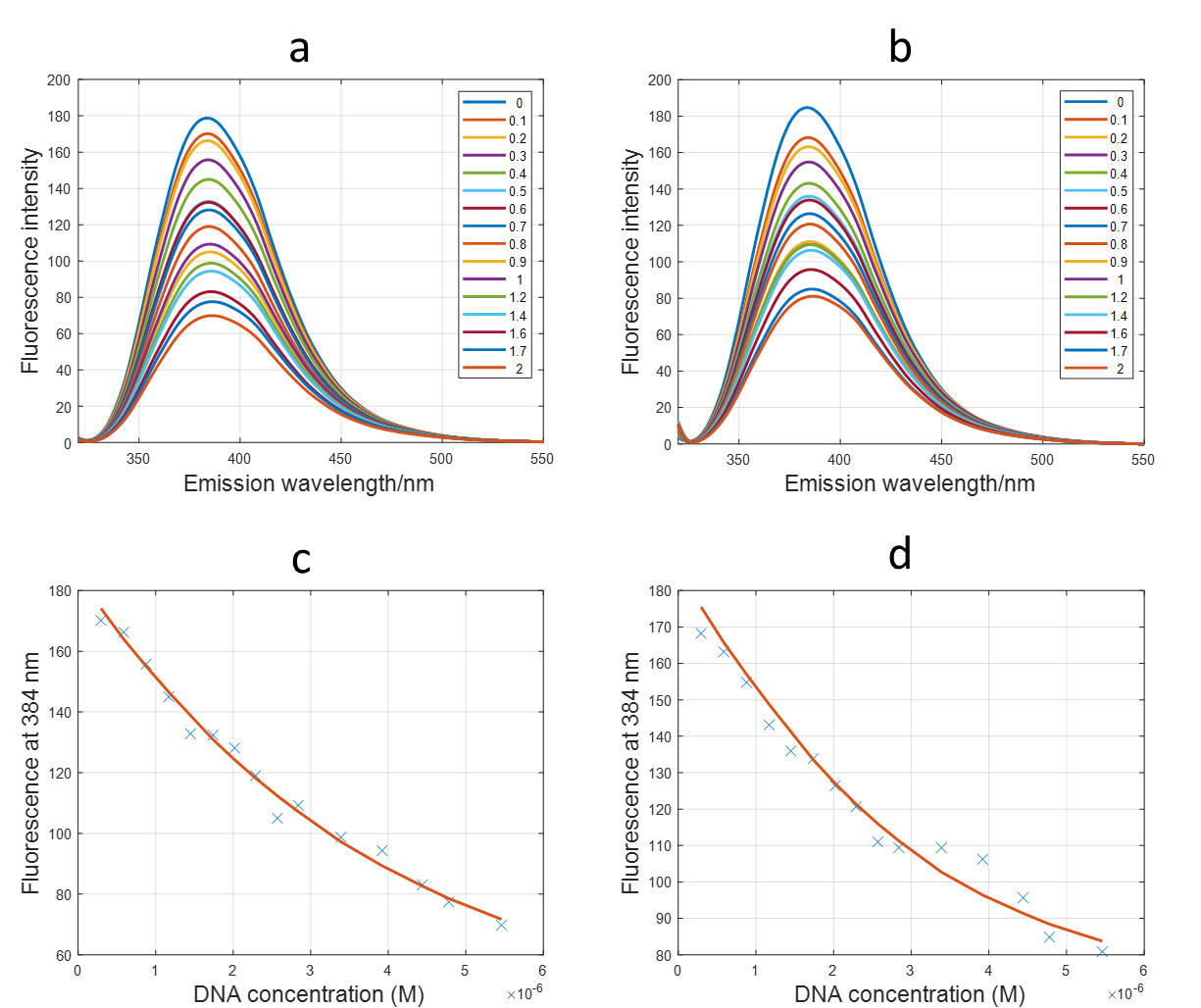


**Figure S5.** Spectra recorded along the titrations of ligand **1** with Pu22T14T23 (a) and d(TTAGGGT)_4_ (b) monitored with molecular fluorescence spectroscopy. Numbers in inset indicate the DNA:ligand ratio. Measured (blue symbols) and calculated (red lines) fluorescence signal at 384 nm considering a 1:1 model for Pu22T14T23 (c) and d(TTAGGG)_4_ (d). In both cases, the initial concentration of ligand was 3 micromolar, 25 mM phosphate buffer and 70 mM KCl, 20^o^C.


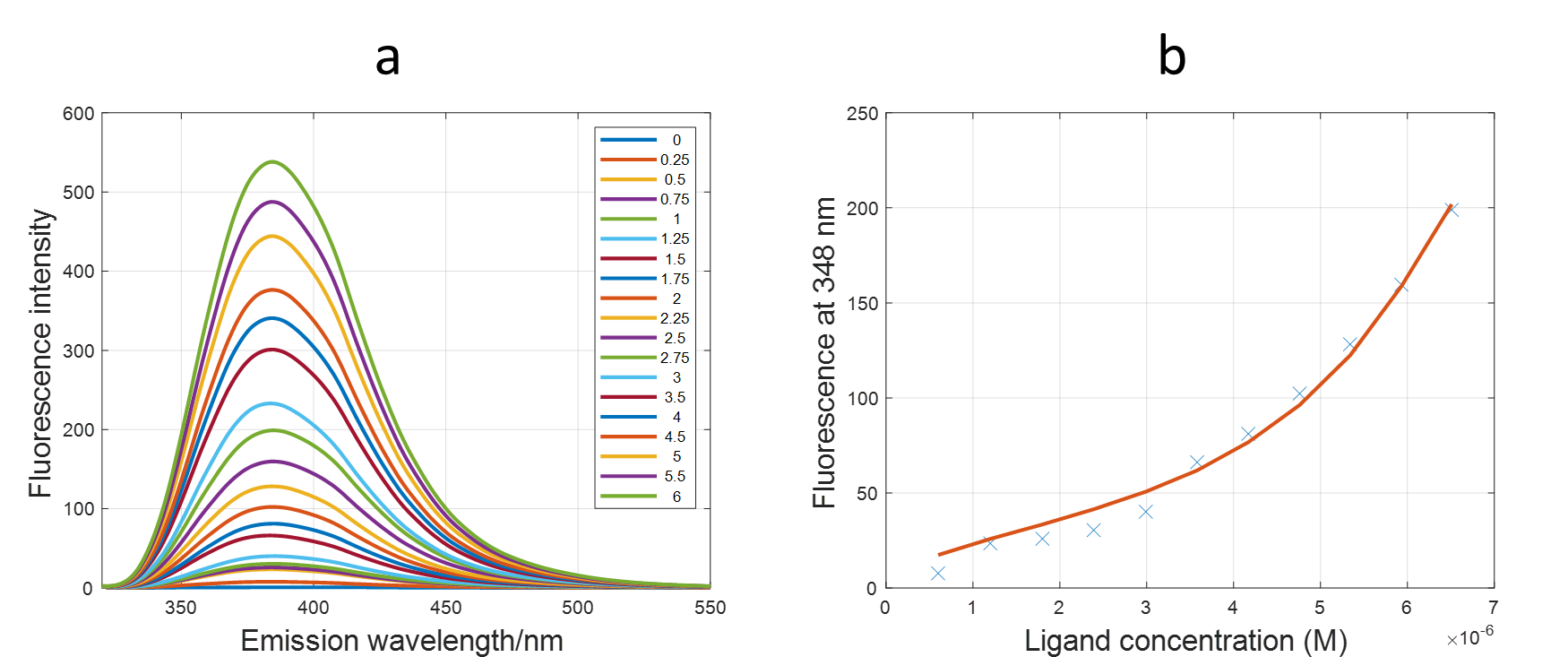


Figure S6. Spectra recorded along the titration of Pu22T14T23 with ligand **1** monitored with molecular fluorescence spectroscopy (a). Numbers in inset indicate the ligand:DNA ratio. Measured (blue symbols) and calculated (red lines) fluorescence signal at 384 nm considering a 1:2 (DNA:ligand) model (b). The initial concentration of DNA was 2.4 micromolar, 25 mM phosphate buffer and 70 mM KCl, 20^o^C.


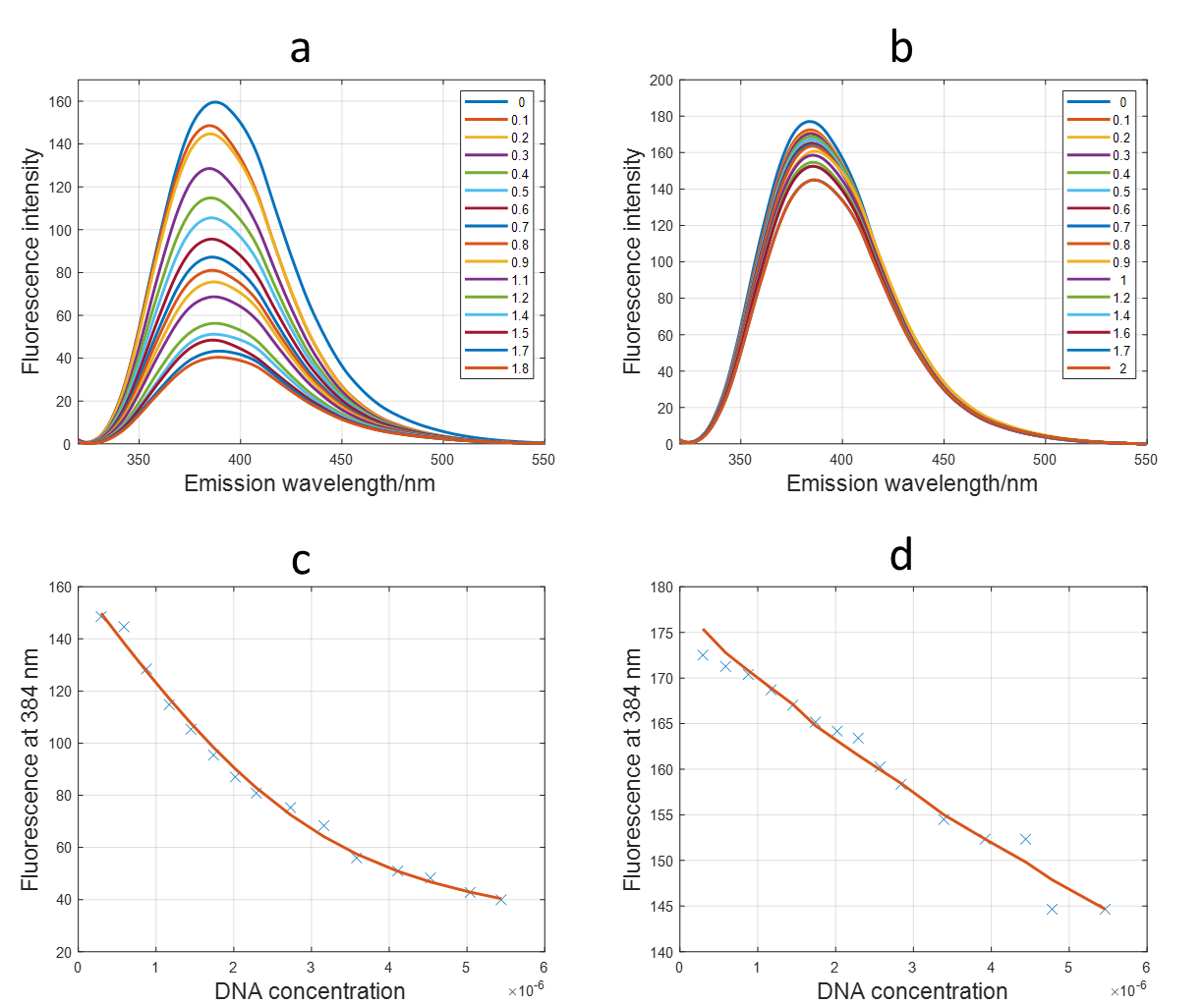


**Figure S7.** Spectra recorded along the titrations of ligand **1** with Pu22T14T23 (a) and d(TTAGGG)_4_ (b) in PBS monitored with molecular fluorescence spectroscopy. Numbers in inset indicate the ligand:DNA ratio. Measured (blue symbols) and calculated (red lines) fluorescence signal at 384 nm considering a 1:1 model for Pu22T14T23 (c) and d(TTAGGG)_4_ (d). In both cases, the initial concentration of ligand was 3 micromolar, PBS buffer 20^o^C.

**Table S1.** Selected ^1^ H chemical shift values for the complex of **1** with d(AAGAATTCTT)_2_ and d(CGTACG)2^a^

| **“AATT”** | H2/H5/CH_3_ | Δδ^b^ | H6/H8 | Δδ^b^ | H1’ | Δδ^b^ |
| --- | --- | --- | --- | --- | --- | --- |
| A1 | n.d. | - | 7.88 | -0.13 | 5.83 | -0.02 |
| A2 | n.d. | - | 8.14 | -0.03 | 5.98 | -0.09 |
| G3 | - | - | 7.65 | -0.09 | 5.78 | +0.03 |
| A4 | n.d. | - | 8.06 | -0.08 | 5.78 | **-0.28** |
| A5 | n.d. | - | 8.14 | -0.03 | 6.18 | -0.05 |
|  |  |  |  |  |  |  |
| T6 | 1.26 | -0.04 | 7.13 | -0.07 | 5.91 | **-0.11** |
| T7 | 1.55 | -0.03 | 7.39 | -0.05 | 6.10 | **-0.11** |
| C8 | 5.63 | -0.05 | 7.59 | -0.07 | 6.03 | -0.08 |
| T9 | 1.70 | -0.10 | 7.51 | -0.08 | 6.19 | -0.04 |
| T10 | 1.78 | -0.03 | 7.50 | -0.13 | 6.24 | +0.02 |
|  | NH |  |  |  |  |  |
| A4T7 | 13.79^c^ | -0.08 |  |  |  |  |
| A5T6 | n.d.^c^ | -- |  |  |  |  |
| G3C8 | 12.39^c^ | -0.07 |  |  |  |  |
|  |  |  |  |  |  |  |
| **“CG”** | H2/H5/CH_3_ | Δδ^b^ | H6/H8 | Δδ^b^ | H1’ | Δδ^b^ |
| C1 | 5.80 | -0.19 | 7.60 | -0.12 | 5.73 | -0.09 |
| G2 | - | - | 7.95 | -0.12 | 5.90 | **-0.13** |
| T3 | 1.42 | -0.17 | 7.29 | -0.07 | 5.89 | **+0.19** |
| A4 | 7.60 | -0.07 | 8.33 | -0.06 | 6.12 | **-0.18** |
| C5 | 5.25 | -0.19 | 7.25 | -0.11 | 5.60 | -0.10 |
| G6 | - | - | 7.87 | -0.09 | 6.02 | -0.17 |
|  | NH |  |  |  |  |  |
| C1G6 | n.d. | - |  |  |  |  |
| G2C5 | 12.64 | **-0.36** |  |  |  |  |
| T3A4 | 13.43 | **-0.19** |  |  |  |  |

^a^ Measured at 15°C in ppm (δ) from external DSS. Solvent H_2_O-D_2_O (90:10 v/v), of 0.1 M NaCl and 10 mM sodium phosphate buffer solution, pH = 7.0; R = 2.0. ^b^Δδ = δ_bound_ – δ_free_; ^c^ Very broad signal.

**Table S2.** Selected ^1^ H chemical shift values for the complex of **1** with d(TTAGGGT)_4_^a^

|  | H1/H2/Me | Δδ^b^ | H6/H8 | Δδ |
| --- | --- | --- | --- | --- |
| T2 | 1.80 | + 0.04 | 7.30 | -0.02 |
| A3 | n.d. | - | 8.39 | 0.00 |
| G4 | 11.44 | - 0.16 | 7.80 | - 0.12 |
| G5 | 11.10 | - 0.11 | 7.62 | - 0.15 |
| G6 | 10.72 | - 0.30 | 7.69 | + 0.01 |
| T7 | 1.70 | +0.10 | 7.48 | +0.14 |

^a^ Measured at 25 °C in ppm (δ) from external DSS. Solvent: H_2_O-D_2_O (90:10 v/v), 25 mM K-phosphate buffer, 150 mM KCl, 1 mM EDTA, pH 6.7. T1 signals are not detected. The ribose protons showing significant shift variations are: T2H1’ Δδ= -0.25. ^b^ Δδ = δ_bound_ – δ_free_.

**Table S3 .** Selected ^1^ H chemical shift values for the complex of **1** with Pu22T14T23.^a^

|  | H1/H2/Me | Δδ^b^ | H6/H8 | Δδ |
| --- | --- | --- | --- | --- |
| T4 | 1.55-1.75 |  | 7.33 | +0.12 |
| G5 | n.d. | - | 8.09 | +0.09 |
| A6 | 7.95 | +0.15 | n.d. |  |
| G7 | 11.40 | **- 0.36** | 7.86 | -0.16 |
| G8 | 11.01 | - 0.21 | 7.60 | -0.12 |
| G9 | 10.70 | +0.10 | 7.71 | -0.04 |
| T10 | n.d. | - | n.d. |  |
| G11 | 11.28 | **-0.43** | 7.83 | 0.04 |
| G12 | 11.34 | -0.16 | 7.86 | -0.06 |
| G13 | 10.89 | -0.16 | 7.68 | -0.18 |
| T14 | 1.77 | -0.15 | 7.65 | 0 |
| A15 | 8.35 | -0.03 | 8.54 | +0.01 |
| G16 | 11.65 | **- 0.35** | 8.15 | +0.04 |
| G17 | 11.05 | - 0.20 | 7.69 | - 0.11 |
| G18 | 10.60 | **- 0.42** | 7.68 | - 0.11 |
| T19 | 2.00 | 0.01 | 7.87 | +0.01 |
| G20 | 10.98 | **-0.30** | 7.84 | - 0.05 |
| G21 | 11.13 | -0.24 | 7.69 | -0.22 |
| G22 | 10.68 | **-0.36** | 7.68 | 0.07 |
| T23 | 1.20 | -0.28 | 7.03 | -0.11 |
| A24 | 7.25 | +0.15 | 7.38 | -0.38 |
| A25 | 7.53 | +0.14 | 7.18 | -0.32 |

^a^ Measured at 25°C in ppm (δ) from external DSS. Solvent H_2_O-D_2_O (90:10 v/v), 25 mM K-phosphate buffer, 70 mM KCl, pH 6.9, *R* = 2.0.

^b^ Δδ = δ_bound_ – δ_free_

**Table S4.** Inter-residue NOE interactions of Pu22T14T23 in the complex with **1**^a^

| NOEs in the tetrads | | | |
| --- | --- | --- | --- |
| *G-tetrad I* | *G-tetrad II* | *Tetrad III* | Other NOEs |
| G7H1-G11H8 | G8H1-G12H8 | G9H1-G13H8 | G13H1-G17H8 |
| G11H1-G16H8 | G12H1-G17H8 | G13H1-G18H8 |  |
| G16H1-G20H8 | G17H1-G21H8 | G18H1-G22H8 |  |
| G20H1-G7H8 | G21H1-G8H8 | G22H1-G9H8 |  |

^a^ Acquired at 25°C in H_2_O-D_2_O (90:10 v/v), 25 mM K-phosphate buffer, 70 mM KCl, pH 6.9; R=2.0
